# Spatial heterogeneity of plant diversity-productivity-carbon nexus in eastern Loess Plateau grasslands

**DOI:** 10.1371/journal.pone.0346057

**Published:** 2026-04-01

**Authors:** Haiyan Pang, Jia Mi, Yao Su, Jincheng Shi, Zonghao Zhou, Jing Shi, Shencong Hu, Xiang Zhao, Kuanhu Dong

**Affiliations:** 1 Shanxi Key Laboratory of Ecological Restoration on Loess Plateau, Institute of Loess Plateau, Shanxi University, Taiyuan, China; 2 Field Scientific Observation and Research Station of the Ministry of Education for Subalpine Grassland Ecosystem in Shanxi, Ningwu, China; 3 College of Environment and Resources Sciences, Shanxi University, Taiyuan, China; 4 College of Grassland Science, Shanxi Agricultural University, Taigu, China; 5 Observation and Research Station for Grassland Ecosystem in the Loess Plateau, Youyu, China; Shandong University, CHINA

## Abstract

This study investigated a grassland ecosystem in the eastern part of the Loess Plateau. Based on the field investigation data from 70 typical grassland sample points in Shanxi Province, we systematically analyzed the regional differentiation characteristics. This analysis revealed the relationship between environmental factors and these key ecological indicators, and established a model for prediction. The results showed that: (1) plant diversity presented a significant altitude-differentiation pattern. The Margalef richness index increased significantly with increasing altitude, whereas the Pielou evenness index decreased significantly. This is closely related to the restricted water and heat conditions, weakened human interference, and changes in the competitive patterns of functional groups in high-altitude areas. (2) There were significant differences in the diversity, biomass, and soil properties among the different grassland types. Temperate shrub grasslands and mountain meadows had high plant diversity and soil carbon-nitrogen ratio. Their complex vegetation structures and refractory litter are conducive to soil carbon sequestration. (3) Soil organic carbon density (SOCd) and net primary productivity above ground (ANPP) show a spatially coordinated distribution pattern. The southern part of the Taihang Mountains and the southwestern section of the Luliang Mountains are high-value areas. The loess hilly area in the northwest of Shanxi Province is characterized as a low-value area due to soil erosion and drought. (4) The regression model constructed based on climatic factors and vegetation characteristics indicated that the average annual temperature and annual precipitation had a significant positive impact on soil carbon content, whereas soil pH and the relative biomass of gramineous plants had a negative effect on species richness. This study revealed the spatial heterogeneity of the structure and function of the grassland ecosystem in the eastern Loess Plateau and its environmental driving mechanisms, providing a scientific basis for regional grassland restoration, carbon management and biodiversity conservation.

## 1. Introduction

Grasslands are widely distributed across the global terrestrial surface, covering approximately 40% of the total area and constituting an indispensable component of terrestrial ecosystems [1]. They play an important role in maintaining ecosystem structure, function, and provision of critical services [2,3]. The Loess Plateau is situated in the arid and semi-arid regions of China and is characterized by severe soil erosion, overgrazing, and frequent droughts. It ranks among the world’s most ecologically vulnerable regions [4,5]. Despite significant achievements in vegetation restoration projects over the past two decades, the grassland ecosystems in this region continue to experience overall degradation pressures [6]. Particularly east of the Luliang Mountains, various grassland types cover more than 32% of the Loess Plateau area [7] however, grazing activities exacerbate the risk of soil and water loss [8]. This region has a complex and diverse topography, encompassing mountains, hills, plateaus, basins, and terraces, with mountains and hills accounting for over two-thirds of the are [9]. This complex terrain, coupled with marked elevational gradients, results in a high degree of spatial heterogeneity in the grassland distribution. Consequently, high-altitude grasslands in mountainous and hilly areas and arid meadows on basin margins simultaneously face the dual challenges of grazing pressure and ecological restoration.

Plant diversity exerts a crucial positive influence on ecosystem function and the supply of ecosystem services [10]. Higher plant diversity not only enhances ecosystem multifunctionality and service capacity but is key to ensuring grassland sustainability [11], thereby increasing ecosystem stability over time and resilience to extreme climatic events [12]. Grassland plant biomass serves as the foundational material supporting animal and microbial communities and is an important indicator for assessing grassland ecosystem functions [3]. Extensive research in the literature has indicated that plant diversity promotes community productivity in both natural and experimental ecosystems [13]. However, human activities and climate change drive global biodiversity loss [14], which may amplify the negative impacts of climate warming on ecosystem functions (such as productivity) and undermine their stability [15]. Grassland productivity is highly sensitive to factors such as climate, soil moisture, and nutrients, and changes in grassland productivity are directly linked to the grassland carbon storage capacity [1]. Therefore, protecting plant diversity and accurately assessing grassland biomass are important for grassland conservation and restoration, forage-livestock balance analysis, ecological monitoring and evaluation, and dynamic resource management [3].

Soil organic carbon (SOC), the primary component of soil organic matter, is a core element that in maintains fundamental ecological functions [16]. Soils represent the largest carbon pool in terrestrial ecosystems, storing significantly more carbon than combined plant and atmospheric carbon pools [17]. Consequently, even minor fluctuations in regard to SOC stocks can substantially influence atmospheric carbon dioxide (CO_2_) concentrations [16]. As a critical component of the global carbon cycle, the soil carbon pool directly affects soil fertility and climate change [18]. Their dynamics are shaped by the combined long-term effects of vegetation, climate, and human activity. Soil carbon sequestration plays a vital role in mitigating the rising atmospheric CO_2_ concentrations [19]. In ecologically fragile regions such as the Loess Plateau, assessing grassland carbon stocks is particularly important for land management and advancing vegetation restoration [20].

Recent years have witnessed increasingly in-depth research on grassland plant diversity, carbon stocks, and productivity in the Loess Plateau, yielding numerous practical recommendations [20,21]. The study area, located east of the Luliang Mountains within the Loess Plateau, is characterized by typical loess-covered mountainous plateaus and a relatively weak natural environmental baseline, thus leading to significant regional heterogeneity in its grassland ecosystems [22]. This study is based on the field investigation and the remote sensing data of 70 representative sample points in the eastern part of the Loess Plateau, aiming to: (1) Explore the response mechanisms of plant diversity and the relative biomass of plant functional groups to altitude gradients and climatic factors; (2) Analyze the differences seen in biomass allocation and soil physical and chemical properties among different types of grasslands; (3) Explain the interaction pathways among environmental factors, plant traits and soil properties; (4) Reveal the spatial distribution characteristics of above net primary productivity (ANPP) and soil organic carbon density (SOCd) of the grasslands in the eastern Loess Plateau; Meanwhile, prediction models for plant diversity and soil carbon content are constructed in order to simulate their spatial distribution. The results from this study will help deepen our understanding of the functional dynamics of grassland ecosystems on the Loess Plateau and provide data support for regional ecological protection and sustainable management.

## 2. Materials and methods

### 2.1. Study area

The study area is located in the eastern Loess Plateau (34°34′N to 40°44′N; 110°14′E to 114°33′E), characterized by a continental monsoon climate with distinct seasons and significant temperature variations. The region features complex topography where mountains and basins intersect, giving rise to considerable climatic and topographical heterogeneity. The mean annual temperature (MAT) ranges from 4°C to 12°C, with some high mountainous areas experiencing temperatures at or below 3°C or even 0°C. The accumulated temperature ≥10°C ranges from 2250°C to 4460°C. The frost-free period lasts between 120 and 220 days. The mean annual precipitation (MAP) varies from 400 to 650 mm, exceeding 900 mm in certain high-altitude zones. Annual sunshine duration ranges from 2200 to 2950 hours. Based on geographical distribution and variations in MAT and MAP, the study area was categorized into a semi-humid southern region and a semi-arid northern region.

### 2.2. Sampling site establishment

**Fig 1:**
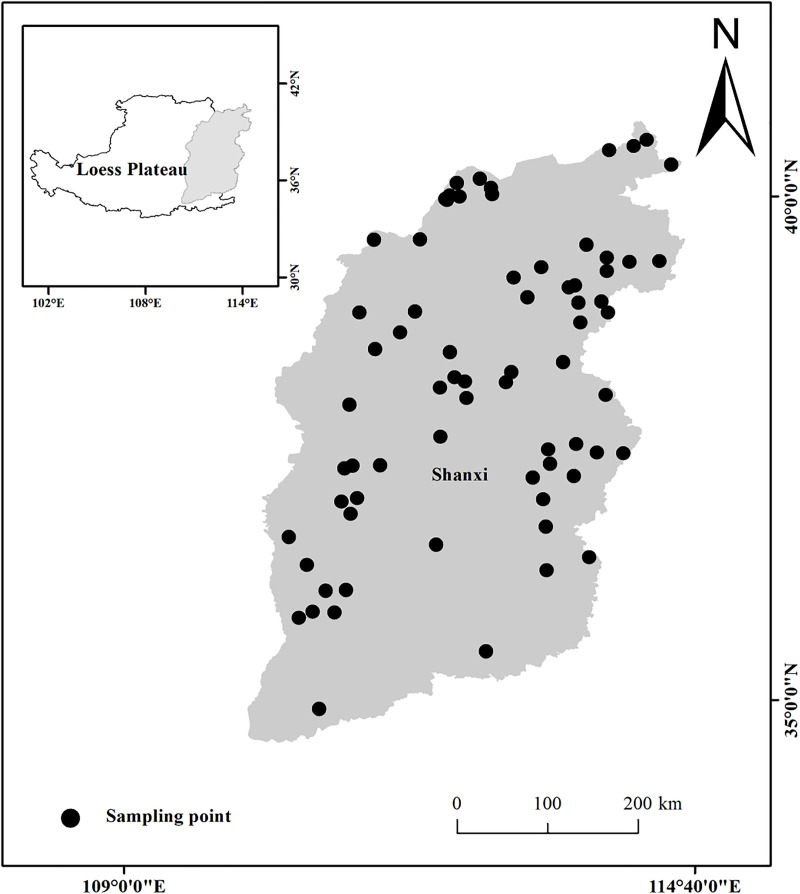
Location of the study area and distribution of sampling points in the eastern Loess Plateau. (Boundary data source from Li et al., 2022, and the Natural Earth. Created using ArcGIS 12.3 (ESRI). The coordinates and elevation data of the sample sites field-measured using GPS).

The survey period was from mid-July to late August 2022, covering 70 sampling points (Fig 1). The study area was distributed across 11 cities and 37 counties in the eastern of the Loess Plateau, involving the four predominant grassland types, namely warm tussock (WT), warm shrub tussock (WST), montane meadow (MM) and temperate meadow (TM). The altitude range was between 300 m and 2500 m.

### 2.3. Data collection and preprocessing

#### 2.3.1. Satellite data.

The grassland distribution data is sourced from: the “ESA WorldCover 10 m 2021 V200 product” dataset, by ESA WorldCover project/Contains modified Copernicus Sentinel data (2021) processed by ESA WorldCover consortium (https://worldcover2021.esa.int) [23]. The boundary map of the Loess Plateau is derived from: Li et al. (2022) [24] and the vector data of municipal administrative divisions in Shanxi Province is from Natural Earth (https://www.naturalearthdata.com). All elevation data and site coordinates (latitude/longitude) were field-measured. Gridded data for MAT and MAP were downloaded from the National Earth System Science Data Center. The map visualizations were presented using ArcGIS 12.3 software (ESRI, Redlands, California, USA).

#### 2.3.2. Field data collection.

Grassland sampling data were collected by field teams from Shanxi University, Chinese Academy of Sciences and Shanxi Agricultural University. At each site, a 100m × 100m representative area was selected. A single 100 m transect was established along the diagonal of this area. Ten 1 m × 1 m quadrats were systematically placed along this transect. Within each quadrat, plant species were recorded, and total community cover, species height, and clump number were measured. Adjacent to each quadrat, a fixed 1 m × 1 m metal frame was used for plant biomass sampling.

Aboveground biomass (AGB) was measured using the harvesting method. AGB components (live plants, litter, and semi-decomposed layer) were collected separately. Standing dead material was included within the litter fraction. Live plants were clipped at ground level, placed in envelopes, and weighed fresh. Litter was carefully collected after clipping live plants, removing adhering fine soil particles, and bagged. The semi-decomposed layer attached to the soil surface was gently scraped using a wool rake, freed from soil particles and debris, and bagged. All samples were oven-dried at 65°C for 48 hours to constant weight and then weighed.

Following AGB harvest within a quadrat, three soil cores were collected along the diagonal of the sample plot and mixed together to form a single sample, which was then placed in a nylon bag with a pore size of 0.3 mm and this process was repeated five times. Each core was separated into seven depth intervals (0–5 cm, 5–10 cm, 10–20 cm, 20–30 cm, 30–50 cm, 50–70 cm, and 70–100 cm). Roots were washed in the laboratory, oven-dried at 65°C for 48 hours to constant weight, and weighed.

Soil samples were collected concurrently with BGB sampling from the same three cores per quadrat. Soil from the three cores for each depth interval was thoroughly mixed. Approximately 500 g of the homogenized soil from each depth was placed in a sealed bag, air-dried in the laboratory at room temperature, passed through a 2-mm sieve after removing fine roots and debris, and ground for subsequent soil total carbon (TC) and total nitrogen (TN) content analysis.

Soil bulk density (BD) was measured using the cutting ring method. A soil pit (1.5 m long × 0.5 m wide × 1 m deep) was excavated adjacent to the center of the transect. Using cutting rings (5 cm height, 100 cm^3^ volume), five replicate samples were taken from each of the seven depth intervals (0–5 cm, 5–10 cm, 10–20 cm, 20–30 cm, 30–50 cm, 50–70 cm, 70–100 cm). Samples were bagged, labeled, oven-dried at 105°C to constant weight, and weighed.

### 2.4. Soil physicochemical properties analysis

Soil carbon and nitrogen content were determined using a German Elemental analyzer. Soil organic carbon (SOC) was specifically measured using the potassium dichromate oxidation (external heating with concentrated H_2_SO_4_) method. Soil pH was measured using a pH meter with a soil:water ratio of 1:2.5.

### 2.5. Calculation of diversity index, relative biomass, soil organic carbon density and net primary productivity

Species diversity was analyzed using the Shannon-Wiener diversity index (*H*), Simpson index (*Ds*), Margalef richness index (*Dm*), and Pielou evenness index (*E*). Each index emphasizes a different aspect of diversity:

The Shannon-Wiener diversity index (*H*) is a composite index that incorporates both species richness and evenness (the relative abundance of species), providing a more comprehensive measure of community heterogeneity.


$$\begin{array}{c} H=-\sum_{i=\text{1}}^{S}\left(Pi\times lnPi\right) \end{array}$$ (1)

where *Pi* represents the proportion of individual species. *i* represents the relative density of plant species (the ratio of the number of individual species to the total number of all species).

The Simpson dominance index (*Ds*) measures species dominance, indicating the probability that two individuals randomly selected from the sample will belong to the same species. A higher value indicates lower dominance and greater diversity.


$$\begin{array}{c} Ds=\text{1}-\sum {pi}^{\text{2}} \end{array}$$ (2)

The Margalef richness index (*Dm*) primarily reflects species richness, i.e., the number of species present in a community, while accounting for sample size.


$$\begin{array}{c} Dm=(S-\text{1})/lnN \end{array}$$ (3)

where *S* represents the number of species, and *N* represents the total number of individuals.

The Pielou evenness index (*E*) specifically quantifies species evenness, describing how evenly individuals are distributed among the different species.


$$\begin{array}{c} E=H/lnS \end{array}$$ (4)

Classify plants based on their characteristics and conduct functional group classification. Calculation of functional group relative biomass:


$$\begin{array}{c} RBi=\left(\frac{Bi}{Btotal}\right)\times \text{1}\text{0}\text{0}\% \end{array}$$ (5)

where *RBi* represents the relative biomass of functional group *i*, *Bi* represents the biomass of functional group *i*, and *Btotal* represents the total biomass of the grassland plant community.

The calculation of soil organic carbon density is based on the soil organic carbon content, soil density and soil layer depth to determine the soil organic carbon density (SOCd, g·m^-2^):


$$\begin{array}{c} SOCd=Ci\times \theta i\times Di\times (\text{1}-\delta i)\times \text{1}\text{0} \end{array}$$ (6)

where *i* represents the soil layer, *Ci* is the average content of soil organic carbon in the *i* layer (g·kg), *θi* is the soil bulk density of the *i* layer (g·cm), *Di* is the thickness of the *i* layer of soil (cm), *δi* is the volume percentage of stones with a diameter greater than 2 mm in the *i* layer (%), and based on the relationship between the soil lithology grade and *δ*, the value of *δ* is taken as 0.5%.

Above net primary productivity on the ground (ANPP) is calculated as:


$$\begin{array}{c} ANPP=AB/S \end{array}$$ (7)

where *S* represents the area of the sample plot, and *AB* represents the above-ground biomass.

### 2.6. Variable selection and modeling approach

To establish the predictive model, we first screened the potential explanatory variables. Through scatter plots and Pearson correlation coefficients, we evaluated the relationship between candidate predictors (such as climate factors, soil properties, and biomass of plant functional groups) and target response variables (soil carbon content and Shannon diversity index). For each target variable, predictors that exhibit a significant linear relationship (*P* < 0.05) are included in the consideration range of the subsequent regression model. Prior to constructing multiple linear regression models, we assessed the linearity assumption for each predictor-response relationship. Scatter plots were visually inspected for non-linear patterns. For any potentially non-linear relationships, we compared the fit of linear models against models including a quadratic term using the Akaike Information Criterion (AIC). In all cases, the linear model provided the most parsimonious fit (ΔAIC < 2), supporting the use of linear regression in our analysis. Based on this screening process and ecological principles, we constructed multiple linear regression models: Soil carbon content was modeled as a linear function of mean annual temperature (MAT) and mean annual precipitation (MAP). Similarly, grassland plant diversity (Margalef index) was modeled as a linear function of soil pH, altitude, and the relative biomass of perennial grasses (PG).

Spatial analysis and prediction employ the Kriging interpolation method (a geostatistical approach based on spatial autocorrelation, which only utilizes the spatial structure of the target variable itself for interpolation), taking spatial autocorrelation into account. Ordinary Kriging analysis was performed using a spherical semi-variogram model. This method provides linear, unbiased, and optimal estimates for unsampled locations based on the regionalized variable theory and variogram modeling. In the process of semi variogram modeling, we systematically compared common theoretical models such as Spherical, Exponential and Gaussian. By calculating and comparing the coefficient of determination (R^2^), residual sum of squares (RSS), and cross-validation root mean square error (RMSE) of each model fitting, the spherical model demonstrated the best fitting effect when characterizing the spatial autocorrelation structure of attributes such as soil carbon content and plant diversity in the study area, and could more accurately reflect the spatial variation characteristics and effective range of variables. Therefore, it was identified as a semi variogram model suitable for spatial interpolation in this study.

The formula for Kriging interpolation is as follows:


z^(s0)=∑i=1nλi z(si) (8)

where: *z^(s*_*0*_*)* is the estimated value at the point *s*_*0*_ to be determined, *z(si)* is the attribute value at the known point *si*, *λi* is the weight coefficient of the known point *si*, and *n* is the number of known points used for estimation.

To validate the accuracy of the spatial predictions, the RMSE was calculated, and cross-validation was performed. Lower RMSE values indicate closer agreement between interpolated results and measured values. The RMSE formula is as follows:


$$\begin{array}{c} RMSE=\sqrt{\frac{\text{1}}{N}}\sum_{i=\text{1}}^{N}(Oi-Pi{)}^{\text{2}} \end{array}$$ (9)

where *Oi* represents the observed value, *Pi* represents the predicted value, and *N* is the total number of data points.

### 2.7. Data processing

Data calculation and organization were performed using Microsoft Excel 2016. One-way analysis of variance (ANOVA) followed by LSD post-hoc tests (α = 0.05) were conducted to analyze the differences in vegetation, soil and climate data under 70 sample points and four types of grassland. Linear regression analysis was conducted to explore the statistical relationship between plants and climatic factors, and the specific values and statistical differences of different grassland types in key indicators such as plant diversity, biomass allocation, and soil properties were presented and compared through bar charts. In addition, Pearson correlation analysis was conducted on each indicator, and a visual heat map was generated as the core visualization tool.

Structural equation modeling (SEM) was conducted using IBM SPSS Amos 24.0 to test the hypothesized causal relationships among environmental factors, plant traits, and soil properties. The modeling process followed a theory-driven, stepwise approach: Firstly, based on the existing ecological theories and previous empirical studies, a prior model was constructed (The theoretical basis for the hypothesized causal relationships in the SEM were provided in Supplementary Text S1 in S1 File). The initial model includes all direct and indirect pathways among altitude, climatic factors, plant functional groups, diversity indices, biomass components and soil properties. The model was identified and fitted using maximum likelihood estimation. Model fit was assessed using multiple goodness-of-fit indices: chi-square to degrees of freedom ratio χ^2^/df, Comparative Fit Index (CFI), Tucker-Lewis Index (TLI), and Root Mean Square Error of Approximation (RMSEA). Acceptable model fit was defined as χ^2^/df < 3, CFI > 0.90, TLI > 0.90, and RMSEA < 0.09. Third, model modification was performed iteratively. Non-significant paths (*P* > 0.05) were removed one at a time, starting with the least significant, and model fit was re-evaluated after each removal. Modification indices were also examined to identify potentially important paths that were not initially hypothesized, but only theoretically justifiable paths were added. The final model was selected based on the combination of acceptable statistical fit and theoretical interpretability.

The diversity index was calculated using the vegan package in R language, and a regression multiple linear regression model was constructed through the ggplot2 package and caret package, aiming to establish an explainable and predictable climate-vegetation-soil relationship model. The ggplot2 package ensures that the visualization of regression diagnosis and results reaches publication quality and validates model hypotheses. The caret package implements a strict model validation process (such as cross-validation) to ensure the robustness and reliability of the model, which is crucial for research on spatial prediction based on models. All spatial data were projected to the WGS 1984 UTM Zone 49N coordinate system and resampled to a uniform 10-meter spatial resolution for consistency in processing and analysis. The 2021 10-m resolution China Grassland Coverage Dataset was processed using the “*Extract by Mask*” tool to delineate grassland distribution. The spatial distribution maps of SOCd and ANPP, as well as the model predictions of grassland carbon content and plant diversity, were fabricated using the common Kriging method of the spherical semi-variogram model. The performance of the model was verified through cross-validation.

## 3. Results

### 3.1 Relationship between altitude gradient, climatic factors and diversity and plant functional groups

**Fig 2:**
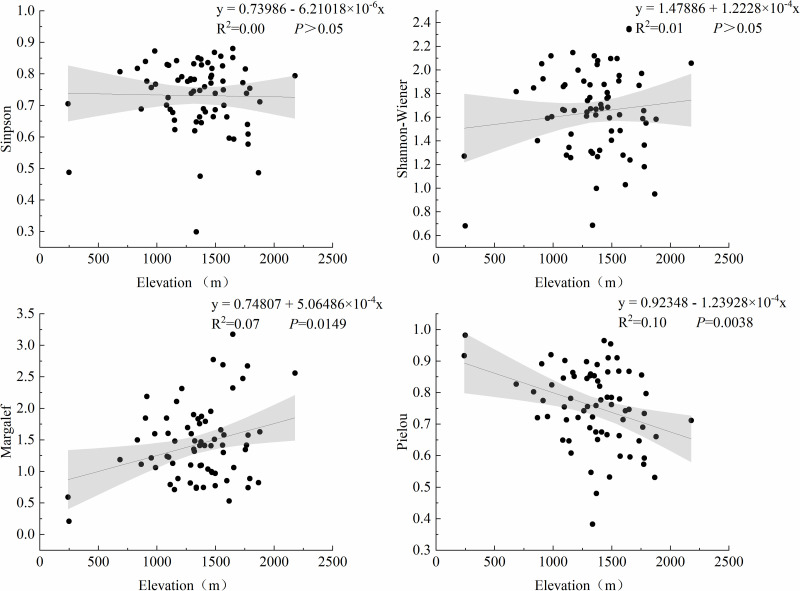
Plant diversity index changing with elevation gradient. The black point is the measured data of the sampling point, the line is the fitting line, and the gray range is the 95% confidence interval.

To explore how environmental gradients shape the structure of grassland communities, we first analyzed the responses of plant diversity and functional groups to altitude and climate (MAT). Our analysis revealed distinct response patterns of plant diversity indices to elevational gradients (Fig 2). The Margalef richness index exhibited a significant positive relationship with elevation (Linear regression: R^2^ = 0.07, *P* = 0.015), indicating increased species richness at higher altitudes. In contrast, the Pielou evenness index demonstrated a significant negative correlation with elevation (R^2^ = 0.10, *P* = 0.004), suggesting reduced community evenness towards mountain summits. Notably, neither the Simpson dominance index (*P* ＞ 0.05) nor the Shannon-Wiener diversity index (*P* ＞ 0.05) showed statistically significant relationships with elevation.

**Fig 3:**
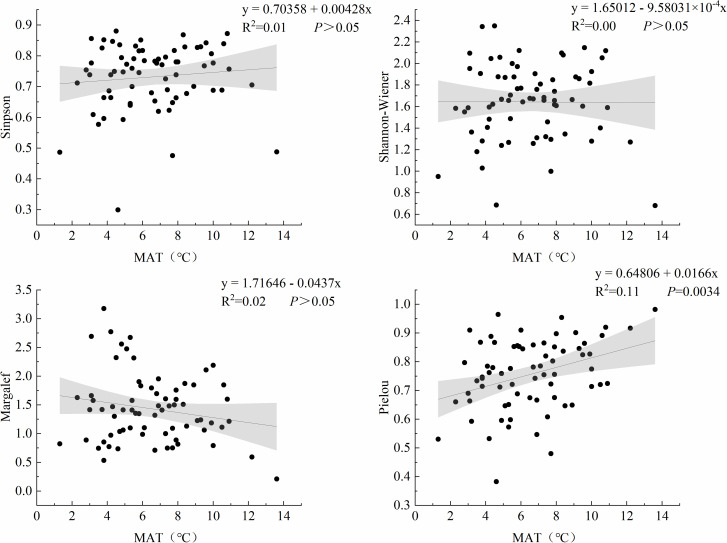
Relationship between MAT and plant species diversity indices. The black point is the measured data of the sampling point, the line is the fitting line, and the gray range is the 95% confidence interval.

The regression relationship between MAT and the species diversity index of grassland plants was analyzed (Fig 3). The results showed that the Pielou evenness index exhibited a significant positive regression relationship with increasing MAT (R^2^ = 0.11, *P* = 0.003), indicating progressively higher community evenness in warmer conditions. In contrast, no statistically significant relationships were detected between MAT and the Simpson dominance index, Shannon-Wiener diversity index, or Margalef richness index (*P* ＞ 0.05).

**Fig 4:**
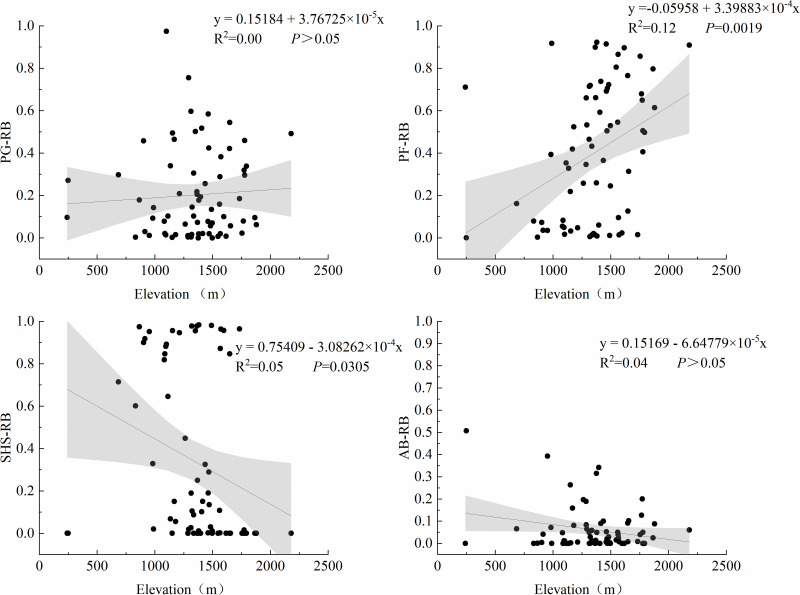
The relationship between elevation and plant functional groups. The black point is the measured data of the sampling point, the line is the fitting line, and the gray range is the 95% confidence interval. (PG-RB, PF-RB, SHS-RB and AB-RB represent the relative biomass of perennial grasses, perennial weeds, shrubs and semi-shrubs, and annual or biennial herbs, respectively, as shown below).

Further analysis examined the relationships between elevation and the relative biomass of plant functional groups (Fig 4). A significant positive regression relationship was observed between elevation and PF relative biomass (R^2^ = 0.12, *P* = 0.002), indicating increased PF dominance at higher elevations. Conversely, elevation exhibited a significant negative regression with SHS relative biomass (R^2^ = 0.05, *P* = 0.031), demonstrating decreased SHS representation with increasing altitude. No statistically significant relationships were detected between elevation and the relative biomass of PG or AB.

**Fig 5:**
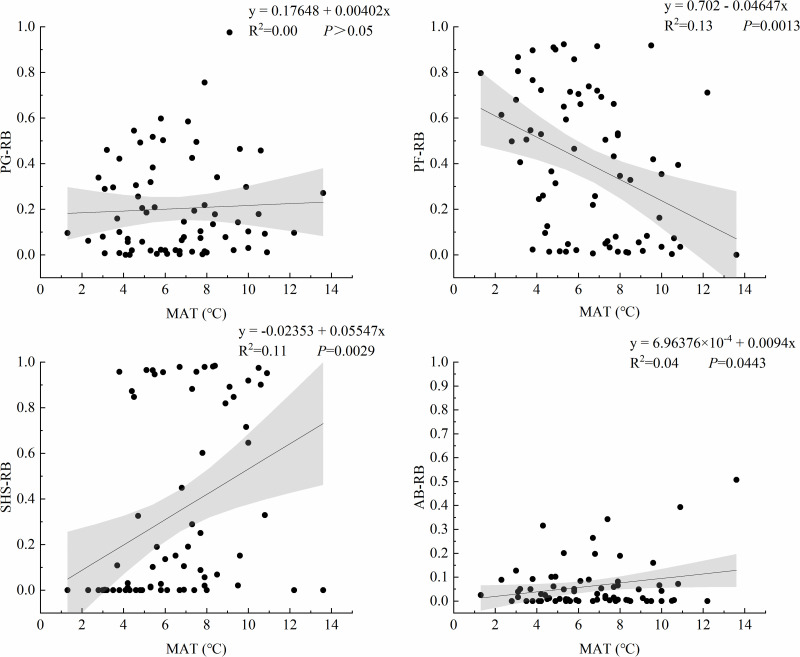
Relationship between MAT and plant functional groups. The black point is the measured data of the sampling point, the line is the fitting line, and the gray range is the 95% confidence interval.

We investigated the relationships between MAT and relative biomass of plant functional groups (Fig 5). PF exhibited a significant negative regression relationship with MAT (R^2^ = 0.13, *P* = 0.001), showing progressively decreasing relative biomass under warmer conditions. In contrast, both SHS (R^2^ = 0.11, *P* = 0.003) and AB (R^2^ = 0.04, *P* = 0.044) demonstrated significant positive regression relationships with increasing MAT. This indicates temperature-driven increases in their relative biomass contributions. No statistically significant association was observed between MAT and PG relative biomass (*P* > 0.05).

**Fig 6:**
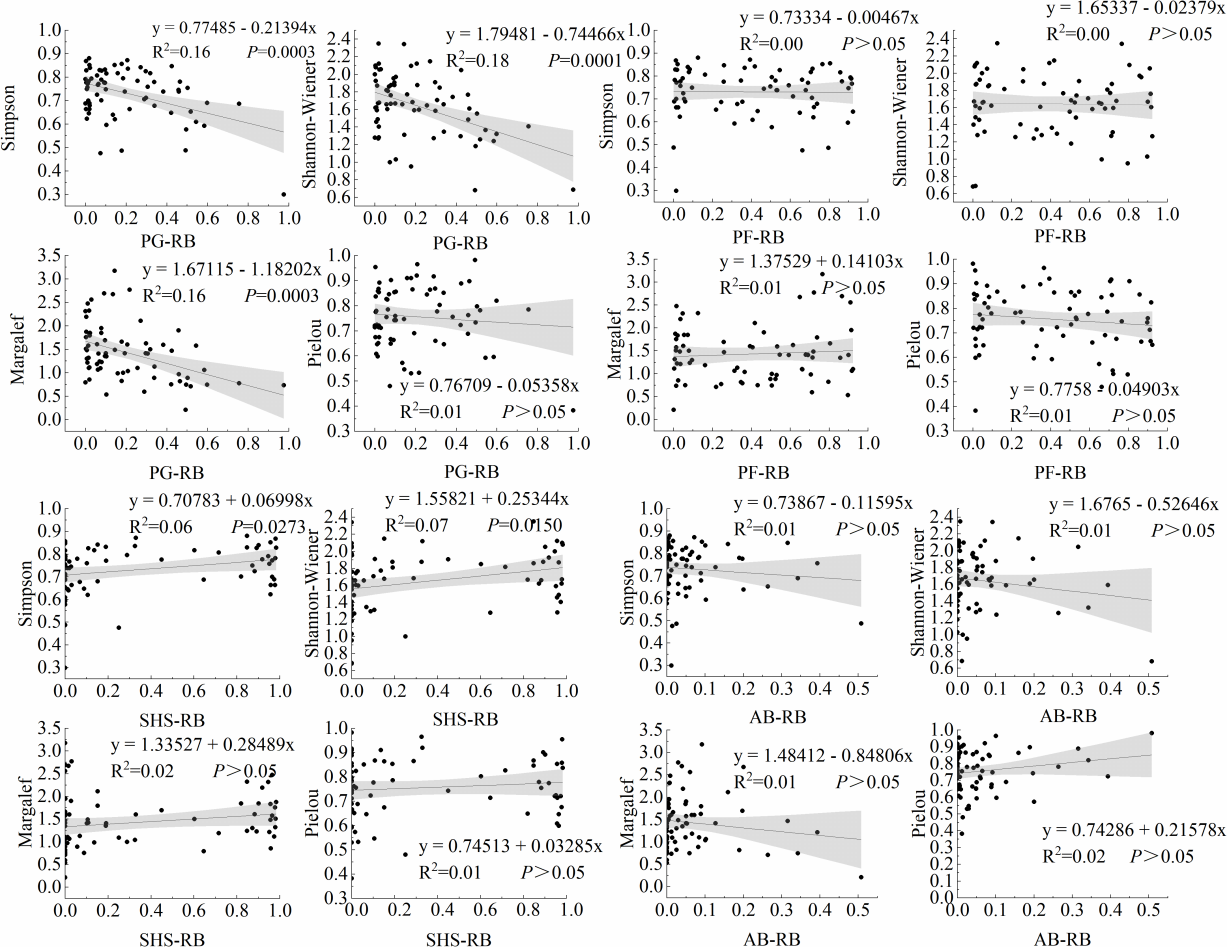
Relationship between plant functional groups and plant species richness. The black point is the measured data of the sampling point, the line is the fitting line, and the gray range is the 95% confidence interval.

Analysis of relationships between plant diversity indices and plant functional group relative biomass revealed significant patterns (Fig 6). We observed significant regression relationships between PG relative biomass and three diversity indices. Simpson index decreased significantly with increasing PG biomass (R^2^ = 0.16, *P* < 0.001). Shannon-Wiener index exhibited a significant negative regression relationship with PG relative biomass (R^2^ = 0.18, *P* < 0.001). Margalef richness index declined significantly with higher PG relative biomass (R^2^ = 0.16, *P* < 0.001). Specifically, our regression model indicated that a 10% increase in PG relative biomass corresponded to an approximate 1.2 decrease in the Margalef index. In contrast, no significant relationship was detected between PG relative biomass and Pielou evenness index (*P* > 0.05).

Significant positive regression relationships were detected between SHS relative biomass and two diversity indices, Simpson index (R^2^ = 0.06, *P* = 0.027) and Shannon-Wiener index (R^2^ = 0.07, *P* = 0.015). No significant relationships were observed between SHS relative biomass and either Margalef richness index (*P* > 0.05) or Pielou evenness index (*P* > 0.05). Similarly, PF and AB relative biomass showed no significant associations with any diversity indices (*P* > 0.05).

**Fig 7:**
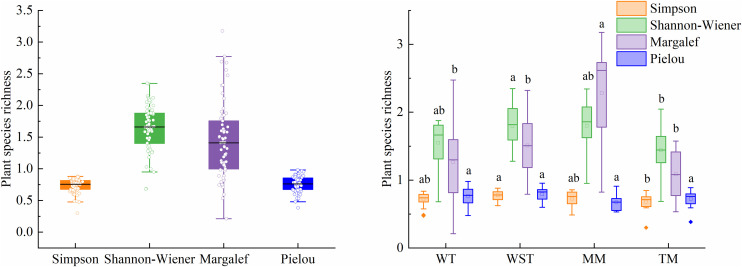
Plant diversity of grassland and different grassland types in the eastern Loess Plateau. Different lowercase letters indicate that there are significant differences in the diversity index under different grassland types. (WT: warm tussock, WST: shrub-tussock, MM: temperate montane meadow, TM: temperate meadow).

The distribution characteristics of plant diversity indices across the study area (Fig 7) showed that the Simpson index ranged from 0.5 to 0.9 (median = 0.7), while the Shannon-Wiener index spanned 1.3 to 1.9 (median = 1.6). Margalef richness index varied between 1.0 and 1.8 (median = 1.4), and Pielou evenness index ranged from 0.5 to 0.9 (median = 0.7). The broader distribution ranges observed for both Shannon-Wiener and Margalef indices suggested higher sensitivity to species composition variations, indicating their potential utility as robust indicators of biodiversity patterns in this study area.

Plant diversity indices exhibited significant variation among grassland types (Fig 7). WT and TM demonstrated comparatively lower diversity values, while WST and MM maintained higher diversity levels. Specifically, WST showed significantly higher Simpson and Shannon-Wiener indices than TM (*P* < 0.05). No significant differences occurred in Simpson and Shannon-Wiener indices between WT and MM (*P* > 0.05). MM exhibited significantly greater Margalef richness than all other grassland types (*P* < 0.05). TM displayed the lowest Margalef richness values. Pielou evenness indices showed no significant differences across grassland types (*P* > 0.05)

### 3.2 Biomass distribution characteristics of grassland

**Fig 8:**
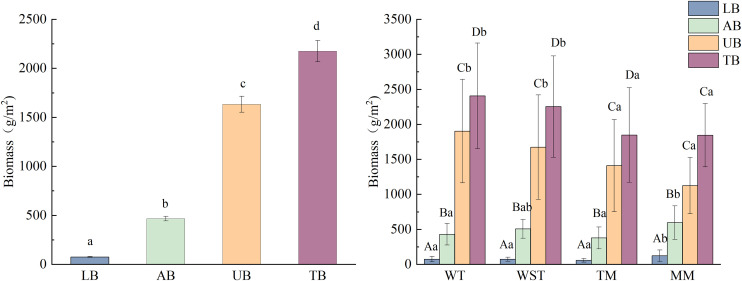
Biomass of grassland and different grassland types in the eastern Loess Plateau. Different lowercase letters indicate that there are significant differences in biomass under different grassland types. (AB: aboveground biomass, UB: underground biomass, LB: litter biomass, TB: total biomass).

After clarifying the environmental drivers of community structure, we further explored how this structure is reflected in the productivity distribution of the ecosystem – that is, the spatial distribution characteristics of biomass. Biomass distribution exhibited significant spatial heterogeneity across the study area. As shown in Fig 8, biomass components followed a consistent hierarchy: belowground biomass (UB)> aboveground biomass (AB)> litter biomass (LB). Belowground biomass (UB) dominated total biomass, accounting for 75.09%. Aboveground biomass (AB) constituted a substantially lower proportion (21.42%). Litter biomass (LB) represented the smallest fraction (3.49% of total biomass).

Analysis of biomass distribution across the four grassland types (Fig 8) showed that MM had the highest aboveground biomass (596.46 g/m^2^), which was significantly greater than that of TM (378.19 g/m^2^) (*P* < 0.05). WST and WT exhibited intermediate values, at 507.25 g/m^2^ and 429.47 g/m^2^, respectively. Belowground biomass was highest in WT (1903.25 g/m^2^), significantly higher than that of MM (1125.15 g/m^2^) (*P* < 0.05). WST and TM recorded belowground biomass values of 1674.04 g/m^2^ and 1409.99 g/m^2^, respectively. Litter biomass was also highest in MM (122.81 g/m^2^), with TM being the lowest (57.95 g/m^2^). WST and WT measured 74.38 g/m^2^ and 72.57 g/m^2^, respectively. No significant differences were observed in total biomass among the grassland types. WST showed the highest total biomass (2332.73 g/m^2^), followed by WT (2181.29 g/m^2^), TM (1788.18 g/m^2^), and MM (1721.61 g/m^2^).

### 3.3 Analysis of soil physicochemical properties of grassland

**Fig 9:**
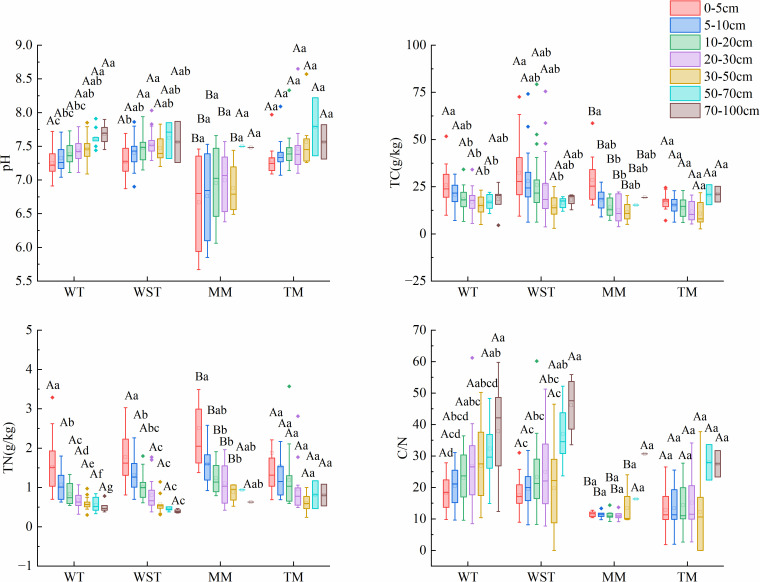
Soil characteristics at different soil layer depths under different grassland types. Different capital letters indicate significant differences in soil characteristics of the same soil layer under different types of grassland, while different lowercase letters indicate that there are significant differences in soil characteristics among different soil layers under different grassland types. (pH: Soil pH, TC: soil total carbon, TN: Soil total nitrogen, C/N: carbon to nitrogen ratio).

The composition and biomass distribution of plant communities may profoundly affect the soil environment. Therefore, we next analyzed the physicochemical properties of soil under different types of grassland to reveal the potential correlation between vegetation and soil (Fig 9). The soil pH of MM was the lowest (6.78 ± 0.30), significantly lower than that of WT (7.38 ± 0.15), WST (7.43 ± 0.19), and TM (7.41 ± 0.27) (*P* < 0.05). Soil TC content was highest in WST (27.73 ± 14.32 g/kg), which was approximately 81.6% higher than that in TM (15.27 ± 5.12 g/kg) (*P* < 0.05). The TC values for WT and MM were 19.49 ± 6.78 g/kg and 18.22 ± 10.09 g/kg, respectively. Soil TN content was highest in MM (1.49 ± 0.62 g/kg), significantly higher than that in WT (0.92 ± 0.31 g/kg) by approximately 62.0% (*P* < 0.05). The TN contents of WST and TM were 1.30 ± 0.55 g/kg and 1.32 ± 1.16 g/kg, respectively. The soil C/N ratio was highest in WST (22.16 ± 7.94), significantly higher than that in MM (13.91 ± 7.31) by about 59.3% (*P* < 0.05). The C/N ratios for WT and TM were 24.01 ± 7.89 and 16.23 ± 8.10, respectively.

Soil properties exhibited significant variation with depth across grassland types. The pH of WT increased from 7.23 at 0–5 cm to 7.70 at 70–100 cm, representing an increase of approximately 6.5%. Both WST and TM showed little pH variation across soil layers, maintaining ranges of approximately 7.35–7.50 and around 7.30, respectively. MM had consistently lower pH values (6.50 at 0–5 cm), with only a slight increase with depth and no significant variation. Soil TC in WT, WST, and MM decreased from 28.90, 35.60, and 32.50 g/kg at 0–5 cm to 16.26, 22.10, and 12.80 g/kg in deeper layers, respectively, representing reductions of 43.7%, 38.0%, and 60.6%. In contrast, TM showed a relatively uniform vertical distribution of soil TC, with 15.80 g/kg at 0–5 cm. Soil TN in WT, WST, and MM decreased from 1.45, 1.75, and 2.40 g/kg at 0–5 cm to 0.54, 0.85, and 0.60 g/kg in deeper layers, with reductions of 62.8%, 51.4%, and 75.0%, respectively. TM exhibited minimal vertical variation in TN, measuring 1.20 g/kg at 0–5 cm. The soil C/N ratio showed distinct depth-dependent patterns among grassland types. In WT and WST, the C/N ratio increased from 22.71 and 19.98 at 0–5 cm to 38.19 (70–100 cm) and 37.34 (50–70 cm), with increases of 68.1% and 86.8%, respectively. In contrast, both MM and TM showed relatively consistent C/N ratios through the soil profile, with vertical variations of less than 15%.

### 3.4 The interrelationships among environmental factors, plant traits and soil characteristics

**Fig 10:**
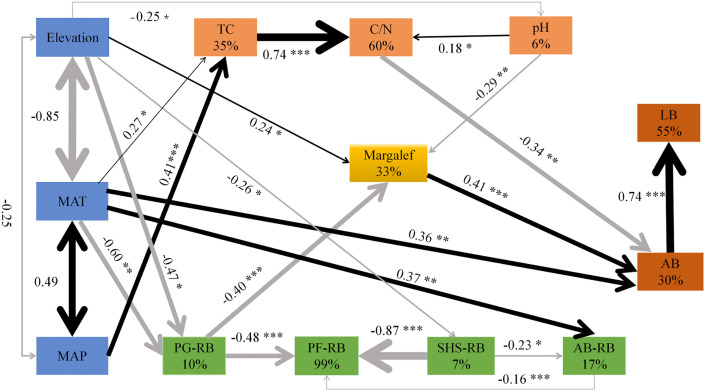
The influence pathway of elevation, climate, vegetation, and soil factors on plant diversity and biomass within the community. Black and gray arrows indicate positive and negative relationships, respectively. Solid or dashed lines indicate a significant (*P* < 0.05) or non-significant relationship. The thickness of the line indicates the size of the path coefficient. The numbers near the path arrows represent the standard path coefficients, with * represented as: ***P < 0.001, **P < 0.01, *P < 0.05. R^2^ is expressed as the proportion of variance explained for each dependent variable. df = 53, χ^2^/df = 1.562, GFI = 0.897, TLI = 0.929, RMSEA = 0.090, *P* = 0.647. Independent samples: n = 70.

To systematically analyze the complex interactions among environmental factors, plant traits and soil characteristics, we constructed a structural equation model and quantified the direct and indirect paths among each element. After repeated model corrections, the final SEM (Fig 10) reached an acceptable fitting level. Elevation exerted a significant positive direct effect on the Margalef richness index (β = 0.24, *P* < 0.01), but significant negative effects on MAP (β = −0.25, *P* < 0.01) and MAT (β = −0.85, *P* < 0.001). The Margalef index showed a significant positive direct effect on AB (β = 0.41, *P* < 0.001), which in turn strongly promoted LB (β = 0.69, *P* < 0.001). Soil pH had a significant positive effect on the soil C/N ratio (β = 0.18, *P* < 0.05), whereas the C/N ratio negatively influenced AB (β = −0.34, *P* < 0.01).

MAP was positively associated with soil TC (β = 0.41, *P* < 0.001). Both MAT and Elevation significantly affected AB, with MAT showing a positive effect (β = 0.36, *P* < 0.01) and Elevation a negative effect (β = −0.47, *P* < 0.05). Additionally, MAT had a significant negative effect on the relative biomass of PG (β = −0.60, *P* < 0.01) but a positive effect on that of AB (β = 0.37, *P* < 0.01). Elevation was negatively associated with the relative biomass of SHS (β = −0.26, *P* < 0.05). Analysis of plant functional groups revealed significant negative effects of PG (β = −0.48, *P* < 0.001), SHS (β = −0.87, *P* < 0.001), and AB (β = −0.16, *P* < 0.001) on the relative biomass of PF. SHS also negatively influenced AB (β = −0.23, *P* < 0.05), while AB negatively affected PF (β = −0.16, *P* < 0.001).

### 3.5 Spatial distribution of SOC density and ANPP

**Fig 11:**
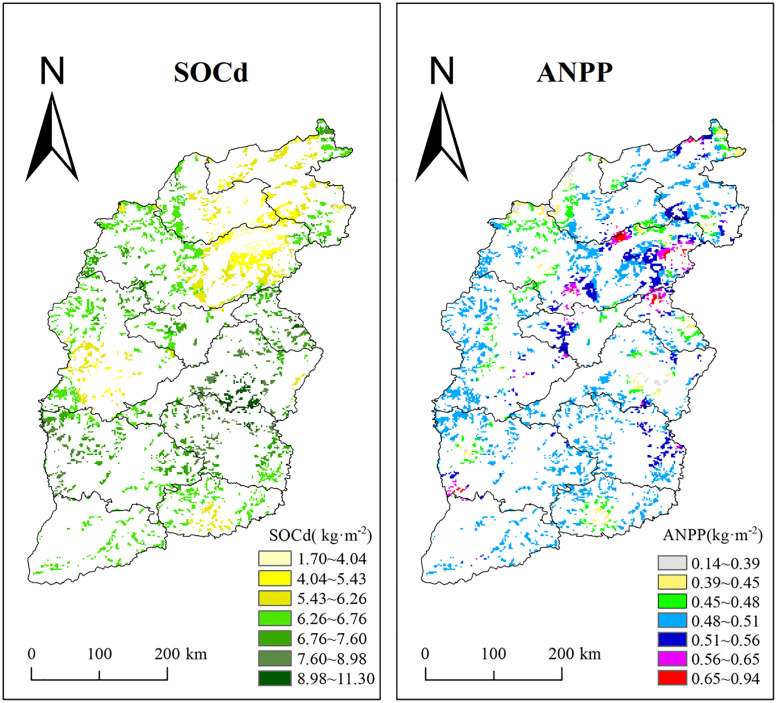
Spatial distribution map of SOCd and ANPP in grassland of the eastern Loess Plateau. SOCd: Soil organic carbon density, ANPP: Above net primary productivity. The blank part belongs to the non-grassland area. Distribution data of vegetation types in China is based from the “ESA WorldCover 10 m 2021 V200 product” dataset. Created using ArcGIS 12.3 (ESRI).

Based on the above local-level mechanism understanding, we extended it to the regional scale and drew the spatial distribution map of soil organic carbon density (SOCd) and above net primary productivity (ANPP) to identify the hotspots of carbon accumulation and productivity (Fig 11, SOCd: RMSE = 1.577; ANPP: RMSE = 0.341). Significant regional variations in both parameters are evident. SOCd values ranged widely from 1.70 to 11.30 kg/m^2^, with a mean value of 6.24 kg/m^2^. The values were classified into seven intervals: 1.70–4.04, 4.04–5.43, 5.43–6.26, 6.26–6.76, 6.76–7.60, 7.60–8.98, and 8.98–11.30 kg/m^2^. Areas with low SOCd (< 4.04 kg·m ⁻ ^2^) were predominantly located in the Loess Plateau region of northwestern Shanxi, including Xinzhou and Luliang cities. These areas are located to the east of the Yellow River and is adjacent to the Mongolian Plateau, which are characterized by nutrient-poor soils, severe soil erosion, and vegetation dominated by sparse shrubs and grasslands. In contrast, high SOCd values (> 8.98 kg·m ⁻ ^2^) were mainly distributed in the central-southern Taihang Mountains and the southern section of the Luliang Mountains, covering parts of Jinzhong, Changzhi, Jincheng, and Linfen cities. These areas benefit from high forest coverage (e.g., natural secondary forests in the Taihang Mountains) and favorable conditions for organic matter accumulation.

The ANPP of grassland areas ranged from 0.14 to 0.94 kg/m^2^ (Fig 11), and was classified into seven intervals: 0.14–0.39, 0.39–0.45, 0.45–0.48, 0.48–0.51, 0.51–0.56, 0.56–0.65, and 0.65–0.94 kg/m^2^. Regions with low ANPP (< 0.39 kg/m^2^) were concentrated in Datong and Shuozhou in northwestern Shanxi, characterized by an arid and cold climate with drought-tolerant herbaceous vegetation. In contrast, high ANPP values (> 0.65 kg/m^2^) were primarily found in eastern Xinzhou and the southern parts of Yuncheng and Linfen in southern Shanxi. These areas benefit from sufficient hydrothermal conditions supported by the Zhongtiao Mountains and the Fen River Valley, and are dominated by deciduous broadleaf forests and farmland ecosystems.

Especially, a certain spatial coupling between high-value zones of ANPP and SOCd was observed. Regions such as the Taihang Mountains (e.g., Changzhi and Jincheng) and the southern Luliang Mountains (e.g., Linfen) exhibited both high SOCd and high ANPP, indicating a synergistic accumulation effect between vegetation productivity and soil carbon storage. Conversely, the ecologically vulnerable Loess Plateau area in northwestern Shanxi (e.g., parts of Xinzhou and Luliang) showed low values in both SOCd and ANPP, highlighting the need for prioritized ecological restoration. These patterns further confirm the crucial contribution of vegetation productivity to soil carbon accumulation.

### 3.6 Model prediction and spatial distribution of grassland carbon content and plant diversity

To quantify the climate and soil drivers of key ecosystem attributes and achieve regional prediction, we established a multiple linear regression model to estimate soil carbon content and plant diversity. To construct a soil carbon content prediction model with a clear mechanism and applicable to regional scales, we selected MAT and MAP as core independent variables (Table 1). This choice is based on the following dual considerations: Firstly, from an ecological perspective, temperature and moisture are the two ultimate climate drivers that regulate soil carbon dynamics. MAT mainly regulates the rate of carbon decomposition by influencing the activity of soil microorganisms, while MAP simultaneously controls the net primary productivity of vegetation (carbon input) and soil moisture (which affects the decomposition environment). Secondly, the preliminary analysis results of this study provide direct support for this choice. The structural equation model (Fig 10) clearly reveals that both MAT and MAP have significant positive direct effects on TC. Therefore, based on the classic theoretical framework of “climate - vegetation - soil carbon” and the empirical evidence of this study, we adopted MAT and MAP to construct a multiple linear regression model of soil carbon content. The regression equation is given as:

**Table 1:** Summary of soil carbon models.

Items		Value	
**Fitting effect**		R^2^ = 0.3465	Durbin-Watson = 1.668
**Variance test**		F = 17.760	*P* < 0.001
**Regression coefficient**	**Constant**	C = −21.845	*P* = 0.013
	**MAT**	b_1_ = 1.261	*P* = 0.000
	**MAP**	b_2_ = 0.08653	*P* = 0.020
**Standardized residual**		−0.0382	–


$$Y=\text{1}.\text{26058}X_{\text{1}}+\text{0}.\text{08653}X_{\text{2}}-\text{21}.\text{84497}$$

where *Y* represents soil carbon content, *X*_1_ denotes MAT, and *X*_2_ denotes MAP.

To predict plant species richness (characterized by the Margalef index), we constructed a model integrating multi-scale driving mechanisms, identifying altitude, PG relative biomass, and soil pH as key independent variables (Table 2). The basis is as follows: Altitude, as an environmental gradient that integrates factors such as temperature and precipitation, is the primary factor driving the macroscopic richness pattern; In this study, the Margalef index was significantly positively correlated with altitude (Fig 2), confirming its role as a large-scale environmental filtering factor. RBPG was extremely significantly negatively correlated with the Margalef index (Fig 10), indicating that perennial grasses reduce community richness through resource competition and are a key variable reflecting local biological interactions. Soil pH, as a core soil factor influencing nutrient availability and microbial activity, although not highlighted in this study, has sufficient literature support for its extensive regulatory effect on community composition. Incorporating it into the model can characterize the impact of the basal soil environment. This model thus integrates three mechanisms: large-scale environmental filtration (altitude), critical functional group competition (RBPG), and basic soil conditions (pH), systematically explaining and predicting the spatial variation pattern of plant richness in the study area. The regression equation is:

**Table 2:** Summary of plant richness models.

Items		Value	
**Fitting effect**		R^2^ = 0.3476	Durbin-Watson = 1.746
**Variance test**		F = 11.720	*P* < 0.001
**Regression coefficient**	**Constant**	C = 5.276	*P* = 0.001
	**Elevation**	b_1_ = 0.0004	*P* = 0.027
	**Relative biomass PG**	b_2_ = −1.123	*P* = 0.000
	**pH**	b_3_ = −0.567	*P* = 0.006
**Standardized residual**		0.1715	–


$$Y=-\text{1}.\text{1}\text{2}\text{3}X_{\text{1}}-\text{0}.\text{5}\text{6}\text{7}X_{\text{2}}+\text{0}.\text{0}\text{0}\text{0}\text{4}X_{\text{3}}+\text{5}.\text{2}\text{7}\text{6}$$

where *Y* represents the species richness index, *X*_1_ and *X*_2_ correspond to the relative biomass of perennial grasses (RBPG) and soil pH, respectively, and *X*_3_ represents elevation.

**Fig 12:**
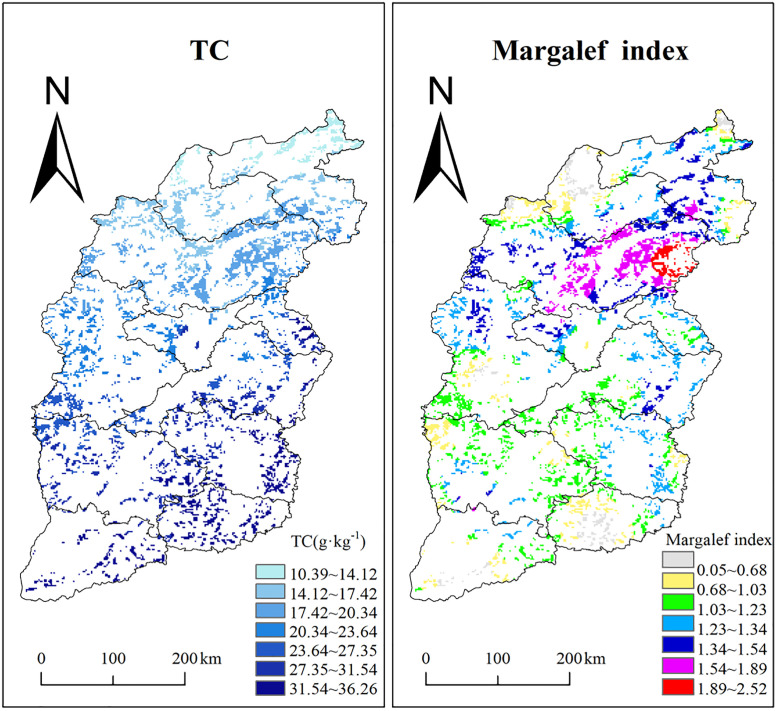
Predicted spatial distribution pattern of TC and Margalef index in grassland of the eastern Loess Plateau. The blank part belongs to the non-grassland area. Distribution data of vegetation types in China is based from the “ESA WorldCover 10 m 2021 V200 product” dataset. Created using ArcGIS 12.3 (ESRI).

Using the above model, we ultimately drew the predicted distribution maps of total soil carbon and plant species richness in the entire study area, visually demonstrating their spatial heterogeneity and providing a reference basis for ecological management (Fig 12, TC: RMSE = 0.005; Richness: RMSE = 0.125).The spatial distribution of TC ranged from 10.39 to 36.26 g/kg, while the species richness index varied between 0.05 and 2.52.

Soil carbon content decreased gradually from southeast to northwest. Low-value areas (10.39–14.12 g/kg) were concentrated in the Loess Plateau region of northwestern Shanxi, including Xinzhou and northern Luliang cities, where severe soil erosion and sparse vegetation cover (primarily grasslands) limit organic matter inputs. High-value areas (31.54–36.26 g/kg) were mainly located in the southern Taihang Mountains (Changzhi and Jincheng cities) and the central-southern Luliang Mountains (Linfen city).

Species richness was generally higher in Xinzhou city, while lower values were observed in the northwest and southeast. Low-richness regions (0.05–0.68) were predominantly distributed in the Datong Basin (Yanggao and Tianzhen counties, Datong city) and Pinglu District (Shuozhou city) of northern Shanxi. These areas are characterized by an arid climate and vegetation dominated by drought-tolerant herbs (e.g., *Stipa* spp. and *Artemisia* spp.). Additional low-richness regions included the Zhongtiao Mountains (in Yuncheng city) and parts of Jincheng city. High-richness areas (1.89–2.52) were primarily found in the eastern Wutai Mountain region (eastern Xinzhou city), which benefits from a humid climate and abundant water availability.

In summary, our results reveal the complete chain from altitude and climate-driven community structure to vegetation-soil interaction, ultimately influencing regional carbon distribution and diversity patterns, and clarify the multi-scale regulatory mechanisms of grassland ecosystem functions in the eastern Loess Plateau.

## 4. Discussion

### 4.1 Reversed elevational diversity pattern and its drivers in eastern loess plateau grasslands

Species diversity and biomass play crucial roles in ecosystem functions [25]. In this study, based on data from 70 grassland plots in the eastern Loess Plateau, we observed a significant increase in regard to species richness (Margalef index) with elevation (Fig 2). This finding contrasts with global meta-analyses reporting general declines in diversity at higher elevations [26], but aligns with the patterns observed in some semi-arid, Mediterranean, or heavily grazed mountain systems where intermediate or high elevations harbor peak diversit [27,28]. This “reverse” pattern often occurs in the interaction between pressure gradients and biological interactions. The deviation in our study area might be due to the selective elimination of low-altitude plants by strong human disturbances (e.g., overgrazing), along with the coordinated regulation of high-altitude hydrothermal conditions (MAT and MAP). Our results have indicated that the reversed diversity-elevation pattern within the study area was largely constrained by these environmental factors [29]. Specifically, in semi-arid regions, such as ours, moisture availability often improves with elevation due to orographic precipitation and reduced evapotranspiration, potentially alleviating primary water stress and creating a more favorable niche for species coexistence at mid to high elevations compared to drier lowlands [30]. Elevational gradients provide a perspective for understanding how biological communities respond to varying environments [31], with significant shifts in plant community composition and structure along such gradients [32].

SEM revealed that increasing the elevation significantly reduced both MAT and MAP. Temperature decline notably altered the community resource allocation pattern by suppressing the relative biomass of the PG (Fig 10), making perennial herbaceous plants the dominant functional group at high altitudes and making a significant contribution to species richness [32]. These perennial forbs significantly influence community species richness [33]. In contrast, perennial grasses often exhibit strong competitiveness at lower elevations, where their extensive root systems access more soil moisture and nutrients, thereby reducing plant diversity [34]. Shrubs play important roles in maintaining species diversity, promoting nutrient cycling, protecting seedling regeneration, and facilitating trophic interactions [35]. However, its biomass decreases with increasing altitude (Fig 10), indicating that low temperatures may directly limit its distribution.

Low temperatures in high-elevation regions are often accompanied by decreased pH and reduced organic matter decomposition rates, thus leading to the accumulation of organic acids [18]. The pH was found to be significantly negatively correlated with plant richness (Fig 10). We also found that perennial forbs decreased with increasing temperatures (Fig 5), probably because locally adapted shrubs and perennial grasses are better suited to slightly alkaline conditions, allowing them to outcompete and replace forbs over time. These dominant plants may suppress forb growth by competing for resources such as water and nutrients. In addition to climatic factors, human activity has played a significant role in recent decades [36]：at low altitudes, the population is dense, and the interference from planting and grazing is strong, which may lead to species loss and a decline in community uniformity. High altitudes are less disturbed by human activities, providing more favorable conditions for the preservation and development of plant diversity.

In conclusion, the reversed pattern of species richness increasing with altitude in the grasslands of the eastern Loess Plateau is the result of the combined effects of water and heat conditions, soil properties, dynamics of plant functional groups, and human activities along the altitude gradient differences.

### 4.2 Differential effects of grassland types on biomass allocation and soil properties

Different grassland types show significant differences in plant diversity, biomass allocation and soil properties. These differences jointly reflect the influence of the interaction between vegetation types and soil characteristics on ecosystem functions. The WST and MM supported higher plant diversity, but their driving mechanisms are different. The WST also exhibited a higher soil carbon content and C/N ratio, which were associated with greater litter input and slower decomposition rates. Litter from shrub and grass communities is usually rich in lignin, and the accumulation of such recalcitrant substances promotes the long-term retention of soil organic carbon [37]. Its near-neutral and slightly alkaline soil (pH 7.0–8.3) may further enhance the microbial activity and nutrient cycling, thereby supporting higher diversity. In contrast, the MM soils were neutral to slightly acidic (pH 6.5–7.0), which might suppress perennial grass functional group competitiveness and provide niches for acid-tolerant herbs, contributing to diversity. However, the lower soil carbon content in MM may result from higher decomposition rates or organic matter loss through surface runoff [38].

The belowground biomass of the WST, WT, TM and MM plants accounted for 79.1%, 74.3%, 76.4% and 61.0% of total plant biomass, respectively (Fig 8). This indicated that they had well-developed root systems. This might be a way for these plants to adapt to dry conditions and better take in water and nutrients. The litter biomass was consistently low across all grassland types, accounting for less than 10% of the total biomass. Moderate grazing may accelerate litter decomposition by directly breaking down dead material and slowing microbial activity under dry conditions as well as indirectly improving plant diversity and litter quality via reduced competition and elevated urease activity [39].

MM had a lower soil pH and higher nitrogen content (Fig 9). This could be linked to higher plant diversity and more dead plant matter falling to the ground. This type of positive feedback helps maintain soil fertility and ecosystem productivity. Increased nitrogen deposition modifies key soil properties, including available nitrogen and pH. These changes directly shift the microbial community structure and activity, consequently effects on nitrogen cycling [40]. Furthermore, elevated soil carbon storage driven by a greater plant diversity can supply the energy required for carbon-intensive microbial metabolism and upregulate the expression of the functional genes involved in the nitrogen cycle, ultimately enhancing soil nitrogen turnover rates [41]. The high carbon-to-nitrogen ratio in the WST suggests that the organic matter in the soil broke down slowly. This may be because the dead plant material from shrubs contained more lignin. Lignin achieves carbon fixation and nutrient release by regulating the carbon-nitrogen balance and inhibiting litter decomposition, thereby indirectly increasing the soil carbon-nitrogen ratio [42]. Differences in pH and the carbon-nitrogen ratio between soil layers, such as, higher carbon and nitrogen in the top 0–5 cm of soil, reflect how roots spread and how dead plant material builds up at different depths.

### 4.3 The spatial differentiation mechanism and coupling relationship between SOCd and ANPP

SOCd is an important measure of soil carbon storage [43]. ANPP is often used to demonstrate how well grasslands store carbon [44]. Understanding the spatial coupling relationship between SOCd and ANPP is crucial for grassland carbon management [45,46]. The area has complex landforms, including the Taihang, Luliang, Hengshan, and Wutai Mountain ranges, as well as several basins such as Datong, Xinzhou, and Taiyuan [47]. In the eastern Loess Plateau, this relationship exhibited significant spatial heterogeneity (Fig. 11). Our analysis identified two dominant and contrasting patterns: a synergistic positive coupling in the southeast and a decoupled negative relationship in the north. The mechanisms underlying this differentiation are primarily driven by regional disparities in climate, land use, and soil conditions.

In the southeastern plateau, a synergistic positive correlation between SOCd and ANPP was observed (Fig 11), primarily driven by favorable climatic conditions and restorative land use policies. It has also carried out “Grain for Green Project” [48]. These have turned some cropland into grassland, which has been mainly used for growing plants, resulting in less grazing or human disturbance. Higher rainfall (>550 mm MAP) and thicker humus layers in the southeast also helped to organic matter build up (SOCd increased by approximately 1.2 kg/m^2^) (Fig 11). This synergy therefore suggests that in these regions, management practices aimed at enhancing vegetation productivity (e.g., sustainable grazing and planting of productive perennial species) are likely to concurrently boost soil carbon sequestration, offering a dual benefit for ecosystem services. However, the soil nutrients there may still be low. The soil pH affects plant growth and nutrient uptake. Alkaline soils may reduce nutrient availability, thereby limiting plant productivity [49].

Conversely, a decoupling effect “characterized by high ANPP but low SOCd” was identified in northern areas such as Xinzhou and Shuozhou. This negative relationship stems from a climatic regime that differentially constrains carbon storage versus productivity. The temperate continental climate with limited rainfall restricts plant growth and organic matter decomposition, leading to low SOCd [50]. However, suitable temperatures and large day-night temperature differences support crop photosynthesis and nutrient storage, which increase productivity [51]. This decoupling phenomenon reveals a potential risk: even if the annual productivity is at a relatively high level, it may not guarantee the long-term accumulation of soil carbon [52], especially when the biomass is mainly composed of annual plants with a low litter mass and rapid cycling characteristics, or when the environmental conditions are conducive to the rapid decomposition of organic matter. We also observed very low ANPP along the northwestern border of Xinzhou and Shuozhou near Inner Mongolia. This may be because the Yellow River carries a large amount of sediment throughout the area, which causes soil erosion and reduces plant growth. For carbon management, this spatial decoupling implies the need for targeted strategies in high-ANPP, low-SOCd areas, interventions should focus not only on maintaining productivity but also on enhancing carbon stabilization (e.g., by introducing deep-rooted perennials, reducing tillage or soil disturbance, and applying organic amendments. In low-ANPP, low-SOCd erosion-prone areas, erosion control and foundational vegetation restoration must be prioritized before significant carbon gains can be expected.

### 4.4 Drivers and predictive modeling of soil carbon pools and plant diversity

To clarify the spatial patterns of soil carbon and plant diversity in grassland ecosystems of the eastern Loess Plateau, and to develop predictive models where feasible, we conducted path analysis on an integrated dataset of vegetation, climate, soil, and topography (Fig 10). The results revealed that soil carbon was primarily regulated by climatic factors, with both direct effects from temperature and precipitation and indirect effects mediated through vegetation productivity. These quantified relationships enabled us to construct regression models for predicting soil carbon content. This interaction path, dominated by climate, ultimately manifested as a distinct southeast to northwest decreasing gradient in soil carbon stocks across the region (Fig 12). Specifically, lower-latitude warming and topography-enhanced monsoon precipitation jointly promoted carbon accumulation in the southeast.

For plant diversity (species richness), path analysis identified a different interaction path, where altitude, soil pH, and relative graminoid biomass were the key direct drivers (Fig 10). The complexity of these interactions currently limits the development of robust predictive models for diversity compared to soil carbon (Fig 12). This path-driven diversity pattern resulted in higher concentrations in areas like the Xinzhou-Dingxiang Basin, where favorable topography and soil conditions mitigated environmental stress, whereas the harsh hydrothermal conditions (e.g., drought and cold) in other regions limited diversity.

## 5. Conclusions

Plant diversity in the eastern Loess Plateau grasslands exhibits significant altitudinal zonation. The Margalef richness index increased with elevation, whereas the evenness index decreased significantly. Constrained hydrothermal conditions and reduced human disturbance at higher altitudes promote niche differentiation between cold-tolerant species and shrubs, whereas intensified competition from graminoids reduces diversity in low-altitude areas. These findings have highlighted the regulatory role of plant functional group competition on diversity, suggesting the prioritized conservation of shrub communities at high elevations and implementation of shrub-grass composite planting in degraded low-altitude areas to optimize vegetation configuration.

WST and MM exhibited higher plant diversity (Shannon-Wiener index: 1.6 ± 0.3) and soil C/N ratio (12.3 ± 2.1). Their complex vegetation structures and recalcitrant litter properties contributed to enhanced soil carbon sequestration. In contrast, the WT and TM showed significantly lower biomass and diversity, owing to monodominant competition and grazing pressure. The SOC content was found to be generally low across the region, with pH ranging from neutral to weakly alkaline (7.0–8.3), reflecting the combined effects of inherent soil infertility and grazing disturbance.

SOCd and ANPP showed significant spatial coupling: both were higher in the Southern part of the Taihang Mountains (SOCd > 8.98 kg/m^2^, ANPP > 0.65 kg/m^2^), while the area east of the Yellow River and the transitional zone with the Mongolian Plateau exhibited lower values (SOCd < 4.04 kg/m^2^, ANPP < 0.39 kg/m^2^) due to soil erosion and drought.

Climatic regression models have identified a substantial decrease in soil carbon across the northeastern Loess Plateau, with modeled increases of 1.265 g/kg per 1°C rise in mean annual temperature and 0.087 g/kg per 1 mm increase in precipitation, projecting a continued carbon decline in this region.

## Supporting information

S1 File Supplementary materials: Theoretical basis and supporting analyses for the structural equation modeling. (DOCX)
