# Supplementary material for: Spatial heterogeneity of plant diversity-productivity-carbon nexus in eastern Loess Plateau grasslands
Source: PLoS One. 2026 Apr 1;21(4):e0346057. doi: 10.1371/journal.pone.0346057 (PMC13042719; doi:10.1371/journal.pone.0346057)
Supplement: S1 File — (DOCX) [file pone.0346057.s001.docx]

**Supplementary Materials**

**Theoretical basis and supporting analyses for the structural equation modeling**

Materials and Methods

**Supplementary Text S**1. A supplementary explanation of the theoretical basis for the interrelationships among factors in the process of constructing a SEM.

Theoretical basis of key causal paths：

Elevation→Climate: Elevation exerts direct effects on climatic conditions through the temperature lapse rate (decreasing temperature with increasing altitude) and orographic effects on precipitation.

Climate→Plant traits and diversity: Climatic factors (MAT, MAP) regulate plant growth, community composition and species richness through metabolic and hydrological constraints.

Diversity→Productivity: The positive relationship between species richness and productivity reflects niche complementarity and sampling effects.

Soil pH→C/N: Soil pH affects the carbon-nitrogen ratio by regulating microbial activity and decomposition processes.

Total carbon→C/N: The influence of TC on the C/N ratio operates through two interrelated mechanisms. First, there is a mathematical dependence, as C/N is calculated directly from TC and total nitrogen. Second, ecological mechanisms are at play: higher carbon inputs from plant litter with specific stoichiometric characteristics (e.g., high C/N litter from graminoids) and preferential nitrogen mineralization by microbes during decomposition both contribute to relative carbon accumulation, thereby increasing the C/N.

C/N → Productivity: The soil C/N serves as an indicator of nitrogen availability for plants. A high C/N ratio typically implies nitrogen limitation due to microbial immobilization, which can constrain plant growth and aboveground biomass production.

Functional group competition: Negative paths among plant functional groups represent competitive interactions for resources such as light, water, and nutrients, which shape community composition and biomass allocation.


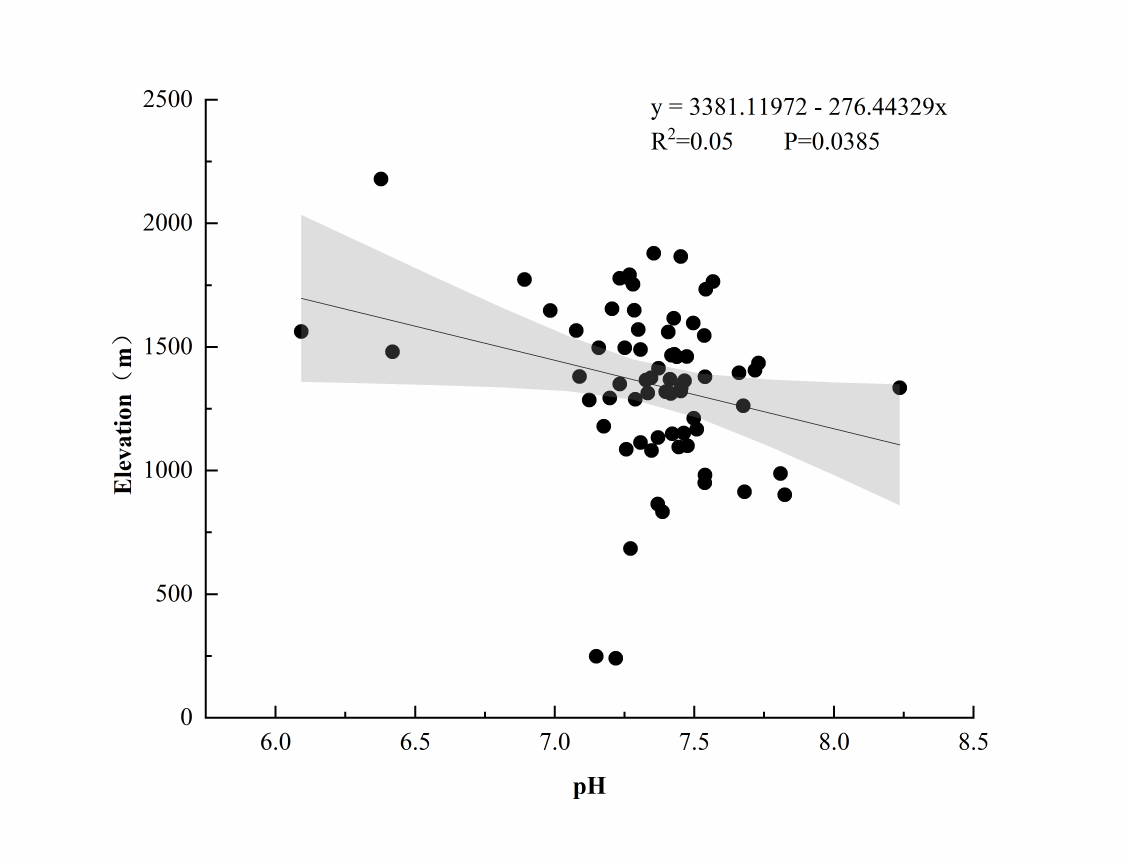


**Figure S1.** Relationship between soil pH and elevation. The black point is the measured data of the sampling point, the line is the fitting line, and the gray range is the 95% confidence interval.


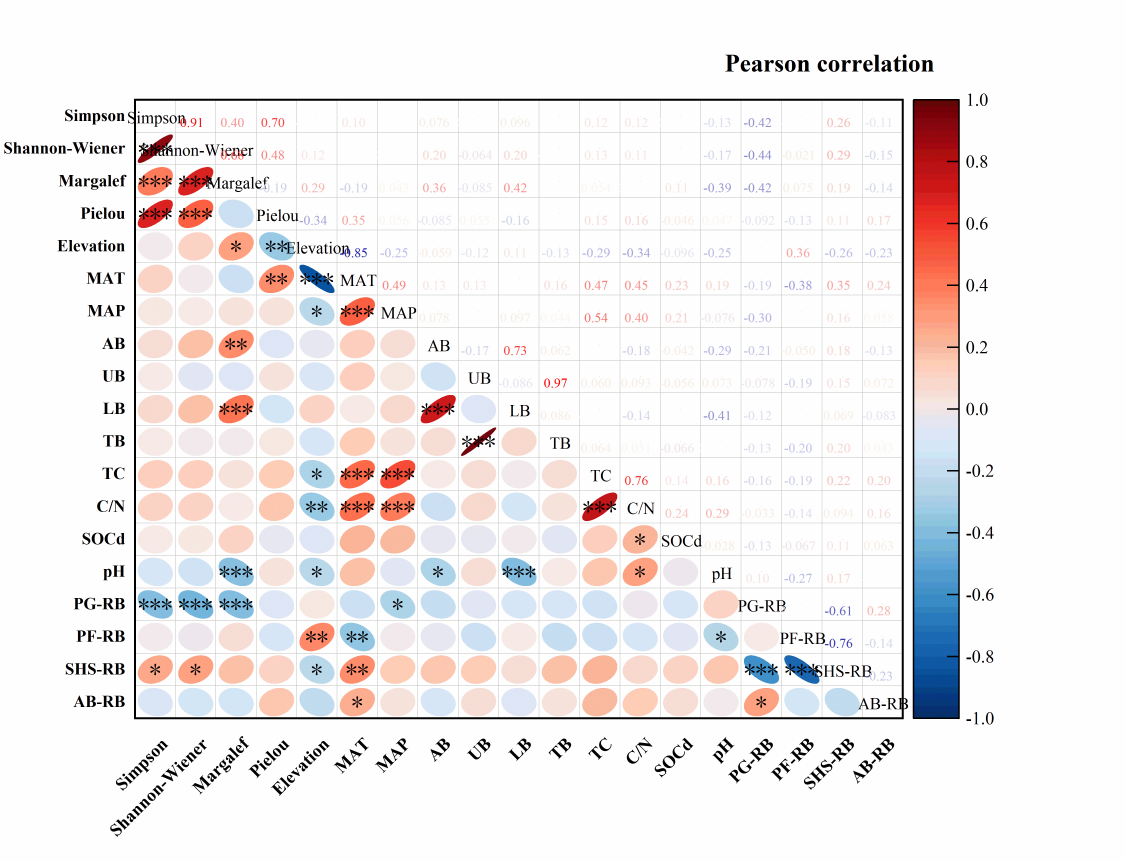


**Figure S2.** Correlation heat map. Plant diversity index, altitude, average annual temperature (MAT), annual precipitation (MAP), aboveground biomass (AB), underground biomass (UB), litter biomass (LB), total biomass (TB), total carbon (TC), soil organic carbon density (SOCd), carbon to nitrogen ratio (C/N), soil pH, plant functional groups (PG-RB, PF-RB, SHS-RB, AB-RB). (Blue represent negative correlations, red for positive correlation, the deeper the color the stronger the correlation. ****P* < 0.001, ***P* < 0.01, **P* < 0.05.)


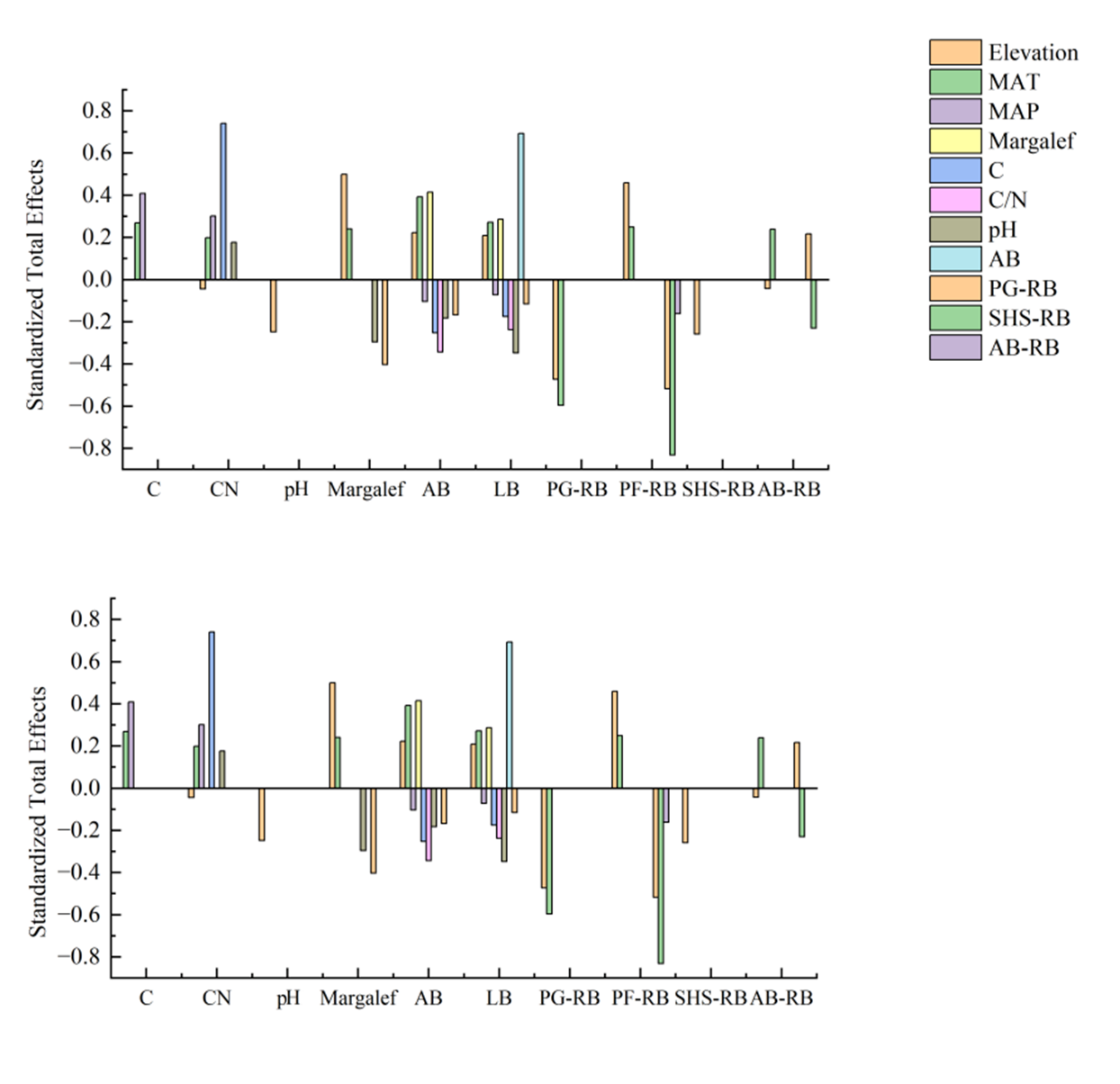


**Figure S3.** The standardized total effect and indirect effect between grassland climate and soil indicators in Shanxi
